# A Distributed Vision-Based Navigation System for Khepera IV Mobile Robots

**DOI:** 10.3390/s20185409

**Published:** 2020-09-21

**Authors:** Gonzalo Farias, Ernesto Fabregas, Enrique Torres, Gaëtan Bricas, Sebastián Dormido-Canto, Sebastián Dormido

**Affiliations:** 1 Escuela de Ingeniería Eléctrica, Pontificia Universidad Católica de Valparaíso, Av. Brasil 2147, Valparaíso 2362804, Chile; enrique.torres.a@mail.pucv.cl; 2 Departamento de Informática y Automática, Universidad Nacional de Educación a Distancia, Juan del Rosal 16, 28040 Madrid, Spain; efabregas@dia.uned.es (E.F.); sebas@dia.uned.es (S.D.-C.); sdormido@dia.uned.es (S.D.); 3 The National Institute of Electrical Engineering, Electronics, Computer Science, Fluid Mechanics & Telecommunications and Networks, 31071 Toulouse, France; gaetan.bricas@ies.univ-montp2.fr

**Keywords:** mobile robot, vision-based navigation, object recognition algorithm

## Abstract

This work presents the development and implementation of a distributed navigation system based on object recognition algorithms. The main goal is to introduce advanced algorithms for image processing and artificial intelligence techniques for teaching control of mobile robots. The autonomous system consists of a wheeled mobile robot with an integrated color camera. The robot navigates through a laboratory scenario where the track and several traffic signals must be detected and recognized by using the images acquired with its on-board camera. The images are sent to a computer server that performs a computer vision algorithm to recognize the objects. The computer calculates the corresponding speeds of the robot according to the object detected. The speeds are sent back to the robot, which acts to carry out the corresponding manoeuvre. Three different algorithms have been tested in simulation and a practical mobile robot laboratory. The results show an average of 84% success rate for object recognition in experiments with the real mobile robot platform.

## 1. Introduction

The current development of robotics has been influenced by the growth of NICT (New Information and Communication Technologies), which has provided the perfect scenario for the confronting of new challenges. In this context, the autonomous navigation of robots based on vision has grown considerably, showing increased interest for some years now.

This interest began some years ago, when researchers presented different approaches for vision-based navigation. For example, in [1], the authors described the development of a system that is capable to calculate the robot position from memorized and currently taken images. While in [2], the authors presented a study on the navigational vision of mobile robots. In this comprehensive and in-depth survey, interesting breakthroughs can be found in approaches that make a distinction between indoor and outdoor environments [3,4,5].

In recent years, navigation based on vision has shown renewed interest in the scientific and educational communities [6,7,8,9] because it has many practical applications in daily life.

Robots that can navigate autonomously are complex systems involving several components that have a common denominator: communication capabilities and the speed of processing and operation. These capabilities are very important because an autonomous robot must have all the necessary information available to make the right decisions at every sampling time [10,11].

Currently, it is common to find robots in the market that integrate cameras, high processing performance and communication capabilities such as Bluetooth or WI-FI, which were not available until recently; examples include the e-puck robot [12], Khepera IV [13], Pioneer 3-DX [14], and TurtleBot3 [15].

These capabilities are important in robot vision-based navigation because robots need to acquire images of their environment, process them, and make decisions to navigate through a scenario [16,17]. In the case of robots that are not capable of performing complex and realistic tasks such as image processing, these images can be acquired with a camera and sent to a server for processing. The server can answer with the orders for the motors of the robot to navigate, or these orders can be calculated in the robot with the results of the image processing [18,19]. This introduces a delay of the network in the control loop, which is an important element that must be considered in these systems. In a local WI-FI network, this delay should not affect the operation of the system. The fact that the processing is done in a server allows another type of hard task, such as the implementation of machine learning algorithms, which require a training stage such as the supervised, unsupervised and reinforcement learning algorithms [20,21,22,23].

The literature shows an effort to add vision-based systems to mobile robot applications. The manuscript [24] presents a system for the detection of persons using cascade classifiers. This approach uses the implementation of HOG (Histogram of Oriented Gradients) feature descriptor and AdaBoost algorithm with a 2D laser and a camera. Similarly, in [25] the development of a vision-based control system for the alignment of an autonomous forklift vehicle is described. The authors implemented Haar feature-based cascade classifiers for detection and heavy spool pick-and-place operation. On the other hand, in [26], the authors presented two systems for detection and classification of traffic signs in real time. The first method is divided into three stages: color segmentation using a threshold, detection using SVM (Support Vector Machine) and classification tree-like and Random Forest. Another approach, like [27], proposes a detection and recognition system for road warning signs with voice notification for autonomous vehicles. It implements Haar feature-based cascade classifiers. Finally, the article [28] describes a navigation system of a robot based on paths following. The method uses a combination of the detection of colors, textures and lines with different classifiers.

During the last few years, a recent effort has focused in the use of Deep Learning for this kind of tasks [29], such as CNN (Convolutional Neural Network), which has become increasingly popular in this field. They can provide a high precision classification for images that are very useful for vision-based navigation in real-time. For example, in [30], the authors present a system capable of navigating and building a map of the environment using an RGB-D camera. Another interesting approach for object detection is Fast R-CNN (Fast Region-based Convolutional Network). This method can efficiently classify the objects using deep convolutional networks. Compared with CNN, it takes an additional sub-network to generate region proposals and it employs several innovations to improve training and testing speed while also increasing detection accuracy [31]. On the other hand, YOLO (You Only Look Once) [32] also has been used for object detection in vision-based navigation in the last years. This algorithm accomplishes object detection via a fixed-grid regression.

As can be seen, there is a wide range of approaches to this topic. After an exhaustive analysis of all mentioned works, we realized that none of them is available for laboratory practices with students, which is one of our main purposes with this research.

This article presents a vision-based autonomous navigation system with machine learning in a laboratory environment. The system has a distributed architecture, which is based on client-server application and it consists of a Khepera IV robot that acquires the images and sends them to a server. On the server, a trained cascade of classifiers processes the images and calculates the linear and angular velocities of the robot to navigate through the indoor experimental environment built in the laboratory. These velocities are sent back to the robot to perform the navigation.

The main motivation for this work is that our Engineering School has implemented a platform for educational and research purposes in the field of mobile robotics [33,34]. In which students can experiment with their own controllers in order to incorporate these concepts in the teaching of robot control. In this sense, experiments on position control, obstacle avoidance, among others, have been implemented with very good results, for example [35,36,37,38].

Now, the purpose is to use the robot’s on-board camera to develop even more challenging experiments that attract the students in an interactive environment. These artificial vision experiments can improve the quality of the laboratory practices, which can mean a step up in the teaching-learning process of mobile robot labs.

The main contribution of this work is to propose the use of advanced computer vision algorithms to perform much more sophisticated and engaging experiments with practical mobile robot laboratories for pedagogical purposes. The motivation of this is to provide much more challenging experiments for the students to improve the quality of the teaching-learning process in this field. A summarized list of contributions of this work is the following: (1) the incorporation of new computer vision capabilities to the practical mobile robot laboratories; (2) to provide much more challenging experiments in an interactive and engaging environment; (3) introduce advanced algorithms for image processing and artificial intelligence techniques for teaching control of mobile robots; and (4) the experiments can be tested in simulation firstly, and after that, they can be implemented in a real and easy-to-use environment in a relatively fast and straightforward way.

The remainder of the paper is organized as follows: Section 2 presents the fundamental concepts related to this article; Section 3 describes the implementation of the experiments in the laboratory; Section 4 shows the results of some test experiments developed in simulation and with the platform; and finally, Section 5 presents the main conclusions and future work.

## 2. Background

The vision-based system consists of the processing of images acquired by the on-board camera. Today, you can find some software libraries to carry out this process, such as Torch3vision, VXL (Vision something Libraries), Library JavaVIS (An Integrated Computer Vision Library for Teaching Computer Vision) [39], LIT-Lib (C++ Computer Vision Library) [40], and OpenCV (Open source Computer Vision library) [41]. After some studies and tests, OpenCV was selected to carry out the image processing. It presents the most complete solutions for the problems that we face processing the images acquired by the robot.

OpenCV is a library of functions for real-time computer vision that was developed by Intel. It is free for use under an open-source license. It has interfaces for C++, Python, and Java. It is very versatile because it can be compiled for Windows, Linux, Mac OS, IOS and Android. It has more than 2500 optimized algorithms that include machine learning, face detection, motion detection, object tracking, 3D object model extraction, finding similar images in a database, augmented reality [42], etc. In our case, its main drawback is that it cannot be executed on the robot. That is why we need to send the images from the robot to a server to process them and calculate the orders for the robot.

### 2.1. Image Segmentation

Different techniques are used to obtain the features of an image. One of the most commonly used techniques is segmentation, which consists of dividing the image into multiple segments (set of pixels) to obtain the information from these segments (also known as super-pixels). The idea is to simplify the image into something that is easier to analyze [43]. This process is commonly used to detect objects and boundaries such as lines, curves, edges, corners, etc. As a result, a set of segments is obtained that is determined by regions with similar characteristics such as intensity, color or texture. The simplest method in segmentation is thresholding, which can create a binary image (black/white) from a color image. If the original image is (x,y), the segmented image is $\left(x^{\prime },y^{\prime }\right)$, which is determined by the threshold U(0<U<255), where 255 is the maximum threshold value for the colors. This operation is defined as follows:

(1) $$\left(x^{\prime },y^{\prime }\right)=255,\;if\;\left(x,y\right)>Threshold$$

(2) $$\left(x^{\prime },y^{\prime }\right)=0,\;if\;\left(x,y\right)<=Threshold$$

**Figure 1:**
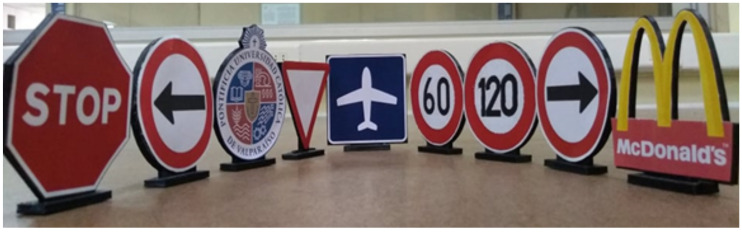
Traffic signals that must be detected.

The value of the threshold is selected depending on the colors that must be grouped to establish, for example, a difference from the background of the image. Figure 1 shows the traffic signals that will be used for navigation.

### 2.2. Cascade of Classifiers

Cascade classifiers are a concatenation of ensemble learning based on several classifiers [44]. Each basic classifier implements the AdaBoost algorithm in a decision-tree classifier with at least 2 leaves [45]. The information from the output of a given classifier is used as an additional input of the next classifier in the sequence [46]. All the classifiers of the cascade are trained with a set of images of the same size called “positive”, which are sample views of the object to be detected and arbitrary “negative” images of the same size.

**Figure 2:**
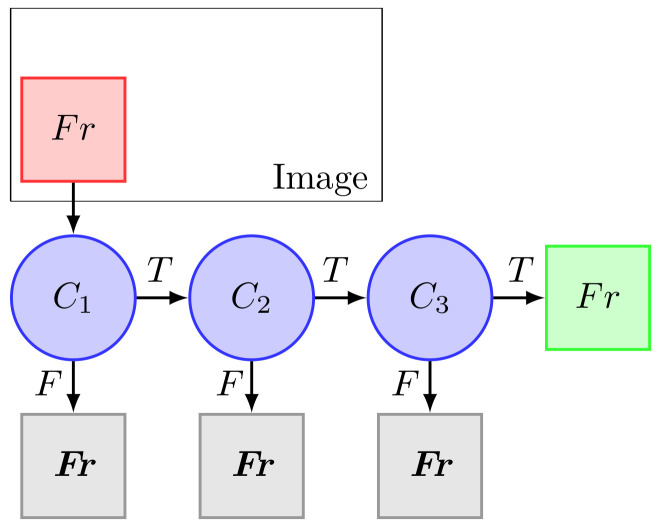
Applying cascade classifiers to the search window on an image.

After the classifier is trained, it can be applied to a search window (of the same size as used during the training) to detect the object in question in the entire frame [47]. This process is repeated until at some stage the analyzed segment of the image is rejected or all the stages are passed. The search window can be moved across the image to check every location for the classifier. The classifier outputs a “*T*” if the region is likely to show the object and “*F*” otherwise. In an image, the extraction of features can be obtained from several methods, including Haar, LBP and HOG. Figure 2 shows this process [48].

The magenta circles represent the classifiers ($C_{1}$, $C_{2}$ and $C_{3}$). The red square represents the frame of the image that is being analysed. If the object is detected, the output of all classifiers is *T*, and the (green square) frame represents this situation. However, if some of the classifier outputs are *F*, the frame is rejected (grey squares), and the object is not detected in this frame.

## 3. Implementation in the Laboratory

### 3.1. Platform Used

**Figure 3:**
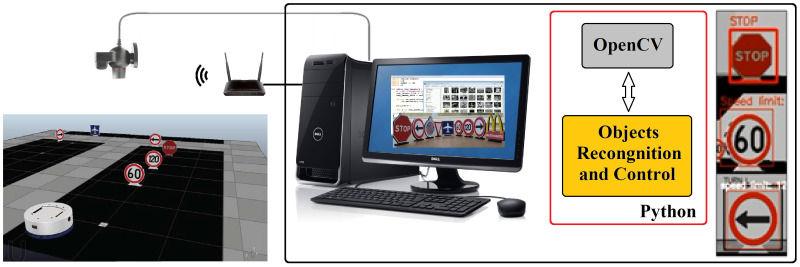
Diagram of the platform used for the experiments.

The implementation of the system is based on the platform developed by the authors in a previous work [33,34,35] with some modifications. The platform has been developed to perform different control strategies in mobile robotics such as path following, tracking control, obstacle avoidance, and formation control. The original platform is based on the IPS (Indoor Positioning System), which provides the absolute position of the robot. For this approach, the position of the robot is not used. Figure 3 shows a diagram of the platform in the laboratory.

The new distributed and vision-based system consists of a client-server application that grants communication and operation of the Khepera IV robot. The server processes the images acquired by the robot and calculates the velocities to control it. The software to perform the video processing has been developed in Python and uses the OpenCV library to work with the images. The wireless communication between the robot and the server is carried out with a WI-FI router. Furthermore, a Play Station 3 USB camera is connected to the server to obtain and overhead view of the experiments. This video signal is used to watch the development of the experiments by the user.

The setup of the platform for the experiments is the following: The dimensions of the arena are 2.0 m × 1.5 m. The dimensions of the robot Khepera IV are 140 mm (diameter) and 58 mm (height). The Dimensions of traffic signals are 26 mm × 26 mm. The Number of traffic signals is 8.

### 3.2. Objects Detection System

**Figure 4:**
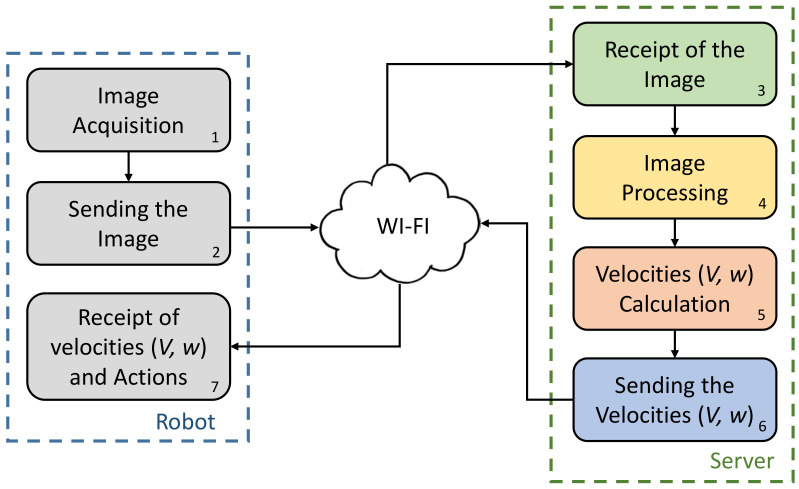
Block diagram of the system.

Figure 4 shows the block diagram of the system. On the left side, the robot is represented by the blue dashed line block.

On the right side, the server is represented by the green dashed line. The server is a PC with the following configuration, Model Nitro AN515-52 a CPU Intel Core i5-8300H, 2.3 GHz, 12 GB of RAM and Ubuntu 19.04.1 64-bits operating system.

The system works as follows, where the numbers represent the order in which the task is executed:

- Block 1: Robot acquires the image. The size of the image is 192 × 116 pixels.
- Block 2: The image is sent to the server using the WI-FI connection. To this end, it has been implemented a standard TCP/IP client-server application in the Khepera and the server. The robot has implemented the application in the programming language C, while the server has implemented the application in Java.
- Block 3: The image is received by the server.
- Block 4: The image is processed in the server.
- Block 5: The linear and angular velocities of the robot are updated using the results of block 4.
- Block 6: The velocities are sent to the robot through the WI-FI connection.
- Block 7: The robot receives the velocities and sends them to the motors.

**Figure 5:**
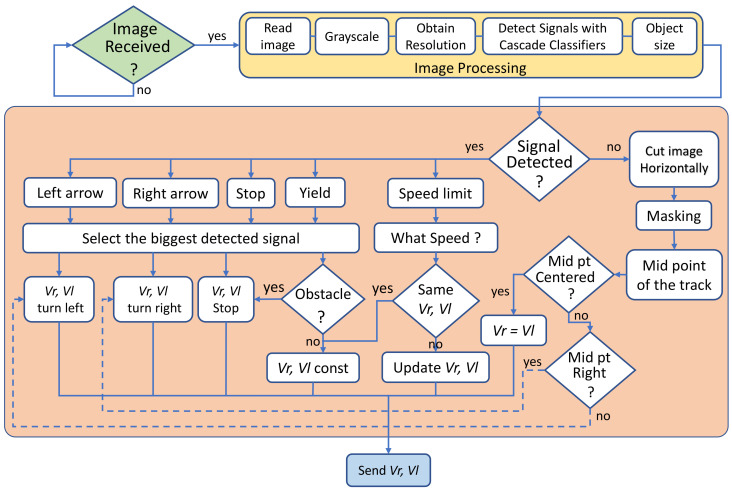
Flow chart of the server side application.

The software that runs the robot is very simple. It consists of two main functions: (1) to acquire the image from the on-board camera and send it to the server, and (2) to receive the velocities from the server and update them in the motors. Note that the image acquisition process does not require extra lights conditions in the lab. On the other hand, the software that runs at the server is more complex because it carries out more difficult tasks, including image processing. Figure 5 shows a simplified flow chart of this application. The tasks are divided into the four blocks of the server side of Figure 4.

The first task is to check if an image has been received. If an image is received, it is processed in the second task (Image Processing) as follows: (1) the image is read, (2) it is converted to grey-scale, (3) its resolution is obtained, (4) traffic signals are detected with the trained cascade of classifiers, and (5) if a signal is detected, its size is obtained. After that, the third task calculates the velocities of the robot from the object detected. At the beginning of this task, the process forks into two main paths: (1) if a traffic signal is detected and (2) if the track is detected.

If a signal has been detected, depending on its type (left arrow, right arrow, stop, yield or speed limit), the velocities are calculated to make the corresponding action. For example: a left arrow $V_{L}$ is equal to 0 and $V_{r}$ is equal to 0.025 m/s, which makes the robot turns to the left. If the detected signal is a stop, both velocities are set to 0. If the signal detected is a speed limit, the signal is processed to determine the corresponding speed. On the other hand, if the object detected is a track, the image is processed by thresholding to determine if the track indicates that it is a straight path or a turn to the left or to the right. In both cases, when the velocities are calculated, they are sent to the robot and the application reverts back to the beginning to wait for other new images.

### 3.3. Implementing Cascade Classifiers

To train the classifiers, existing databases of “positive” and “negative” images can be used, or customized databases can be created. These image training sets can be created using OpenCV. In our case, the “negative” image set was created using the on-board camera of the robot.

**Figure 6:**
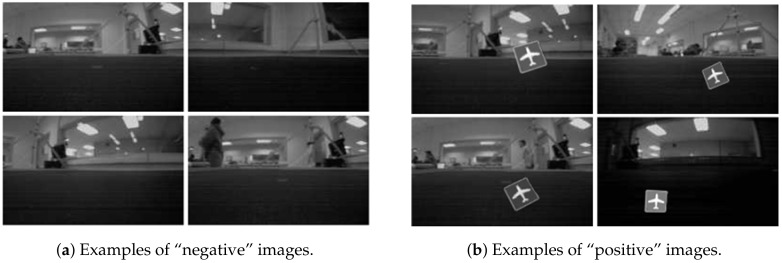
Examples of images acquired by the robot.

Figure 6 shows on the left side, 4 examples of “negative” images obtained by the robot. To provide correct training, these images must not contain the object that wants to be classified as positive. The set of negative images for this experiment was composed of approximately 1400 images. Figure 6 shows on the right side, 4 examples of the positive images acquired by the robot. In this case, the object that wants to be detected is the signal of the airport, which is included in all positive images. In this case, the number of positive images was approximately 1000. With these two sets of images, the OpenCV function “training cascade” was used to carry out the training stage.

The selected classifiers were Haar-like with 5 stages in the cascade. A high number of classifiers in the cascade improves the classification, but the training time can increase exponentially. We selected Haar feature-based algorithm because this classifier showed encouraging performance with high success recognition rates for this experiment.

### 3.4. Application of the Robot

As mentioned before, the programming code of the robot has been developed in Ansi C, which is the programming language that the robot uses. The following code segment shows the pseudo-code of this application. Note that some lines have been omitted for space reasons. Note that, the source code of both applications is available online on GitHub.


1 Import of the libraries 
2 main function
3   // Define variables and Server Socket
4   // Server Socket is listening (192.168.0.111:8080)
5   // Accept connection from an incoming client
6   // Initiate libkhepera and robot access
7   // Open robot socket, store the handle in its ptr 
8   // Initialize the motors with tuned parameters
9   // Receive a msg from client
10  // Client Socket receives message?
11  // Execute received velocities
12  // Close socket
13  // If an erroneous msg received rise an error msg 
14  // If some button is pressed program stops


The first lines define variables and some configurations. Then, the communication socket is created and connected to the server. Through this connection, the robot sends the images acquired by the camera of the robot with the MJPG-streamer application [49]. Then, the parameters of the motors of the robots are initialized. After that, the received message from the computer is processed. Finally, the velocities of the robot received in the messages are sent to the motors.

### 3.5. Application of the Server

The following pseudo-code shows the implementation at the server side. This code has been developed with Python. An important detail is that the server reads the images acquired by the robot using the TCP connection created and the application MJPG-streamer that is running at the robot. Note that some lines have been omitted for space reasons.


1 Import the needed libraries
2 While loop until a key is pressed:
3  # Read the image from the robot
4  # Execute cascade classifiers with the image
5  stops?     # Is the STOP signal?
6  airport?   # Is the airport signal?
7  rArrow?    # Is the right arrow signal?
8  lArrow?    # Is the left arrow signal?
9  # Depending on the detected signal 
10  # its area is calculated
11  # Speed limits are calculated using Flann technique
12  # Time of action of the signal 
13  if time-stop_time is less than 5, then state is equal to STOP
14  else if time - time to turn to Left is less than 5, then state is equal to TURN LEFT
15  else if time - time to turn to Right is less than 5, then state is equal to TURN RIGHT
16  else state is equal to GO
17  if state is equal to GO, then      
18  # Calculate the center of the track
19  # Creating the Mask
20  # Calculate the velocities for each signal            
21  # Velocities converted and send them to the robot
22  # Sending the velocities to the robot
23 if a key pressed, exit


The first lines carried out the variables definition and libraries invocation. Then, the socket is created and connected; and the trained cascade classifiers are loaded. After that, the main loop starts and the image from the robot is obtained and conditioned. Then, the cascade of classifiers is applied to the obtained image. The result of this operation is that only one classifier must be true. If not, the traffic signal is detected with the classifiers, which means that the track must be followed. With the result of the classifiers, the velocities are calculated. Finally, the velocities are sent to the robot and the program waits for a key to finish.

## 4. Experimental Results

In this Section, all the tests that were carried out for the detection of objects and navigation of the robot are shown. The results are represented from the detection of basic signals (arrows of the same color) to more complex signals, which are involved in the tests of the designed classifiers (presented in Section 3), as well as traffic signals and new scenarios. These results reflect the efficiency of this new algorithm, both simulated and with the real robot. The Section describes three experiments. The first experiment shows the difficulties of detecting multiple objects by using traditional image processing. The second experiment describes the advantage of the cascade classifier for recognition of several traffic signals, and finally, the third experience provides the results of the implemented vision-based navigation system to perform an advanced experiment in the real environment.

### 4.1. Experiment 1: Object Detection by Image Processing

To initiate the proposed algorithm, a simulation of a simple scenario was performed using the V-REP simulator and the Khepera IV robot model previously developed by the authors [36,37]. The experimental concept is that the robot can navigate through the arena, detecting the arrows.

**Figure 7:**
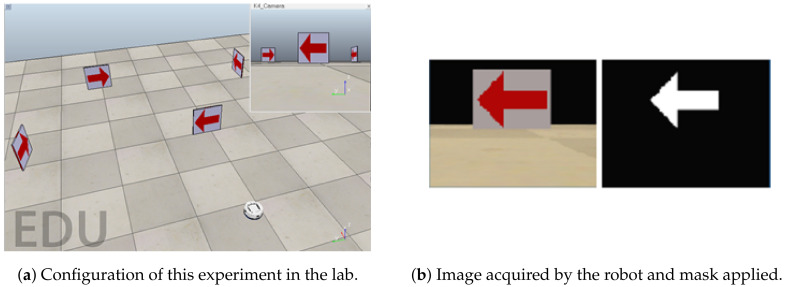
Detection of arrows of the same color in a simulated environment.

This work is based on a previous work of the authors presented in [38]. The robot control algorithm must be able to calculate the corresponding speeds that allow the robot to turn in the correct direction depending on the arrow detected. For the first experiment, the arrows are of the same color to simplify the identification because, using the threshold technique, the arrows can be easily detected. Figure 7 shows the configuration of this experiment on the left side. In the upper right corner of the scenario, the images acquired by the on-board camera of the robot are shown in running time.

Once the image is detected by the robot in the program, red masking is performed, which only displays the characteristics of the red arrow, as shown on the right side of Figure 7. The image on the left is the image acquired by the robot camera, and the image on the right side is the result from the red masking. The image is segmented in such a way that there is a margin where only the color red appears. In that image, there may be several arrows, so the robot determines the largest arrow, since it is assumed that this arrow will be the closest to the robot and thus will have more relevance.

OpenCV provides multiple tools to discern the orientation of an object, including *cv.minAreaRect*, *cv.fitEllipse* and *cv.fitLine*. In our case, *cv.fitLine* was used, which fits a line based on a given set of points, such as a contour. This function provides four variables that can be used to obtain two other variables called *lefty* and *righty*. In the case of arrow detection, if it is oriented to the left, the value of the *lefty* variable is positive, and *righty* is negative. However, if the arrow is oriented to the right, these values change their sign.

**Figure 8:**
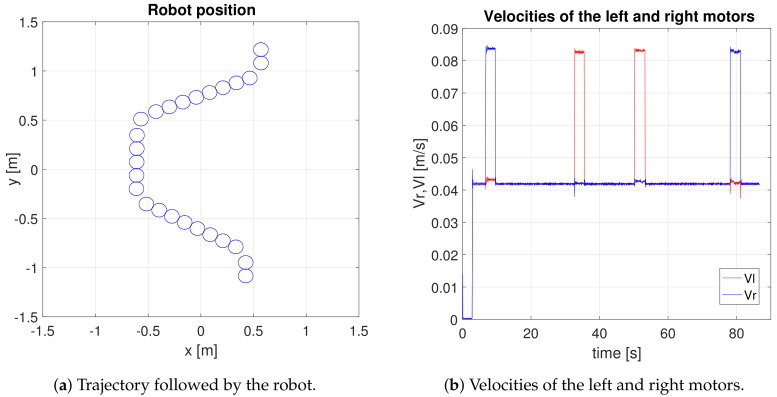
Experiment carried out in simulation environment.

Figure 8 shows the path of the robot on the left side, which indicates that the robot is performing autonomous behaviour. It detects the arrow direction and makes the correct decisions to turn to the right and to the left depending on each arrow.

The right side of this figure shows the speed behaviour of each motor during the experiment. In the first stage, the speed of the right motor is greater than that of the left, so the robot makes a leftward turn. Then, the speeds are equal again. In the second stage, the speed of the left motor is greater, so the robot turns to the right. In the third stage, the robot turns to the right, and in the last stage, the robot turns to the left. In conclusion, the robot detects four arrows and affirmatively avoids them by producing an algorithm to detect and avoid arrows.

**Figure 9:**
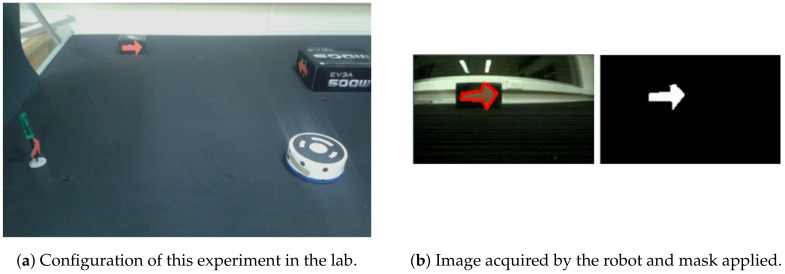
Detection of arrows of the same color in a real environment.

As in the simulated experiment, a test involving the detection of arrows was also carried out with the platform. Figure 9 shows the configuration of this experiment on the left side. The robot is near three red arrows, which are positioned at 80 cm and have an orientation of 90 degrees with respect to each other. As in the previous case, the goal of the robot is to detect these arrows and make decisions to navigate through the scenario.

On the right side of Figure 9, the image acquired by the robot and its corresponding masking of the red color are shown. In the virtual experience, the *cv.fitLine* function worked correctly, but for the real environment the results were not suitable to perform a clearly detection of the orientation of the arrows. The main reason is due that *cv.fitLine* requires a sharp outline or contour (i.e., bounding the shape) of the arrow, which could be however noisy by the changes of illumination in the case of the real environment. This issue makes difficult the detection of the orientation by the *cv.fitLine* function.

Therefore, another technique was used according to the following criterion: An arrow has seven vertices, but two of them are located at the top and bottom of the image; therefore, depending on the location of those points on the x-axis, the robot can determine the orientation of the arrow.

Note that this is a previous stage of the algorithm that we want to implement. This method needs a lot of knowledge (features) about the object to be identified, that the designer of the algorithm has to identify discriminant features (such as borders, colors, etc.) in order to recognize the objects. That is why we have used OpenCV functions and the robot Khepera IV, to show this drawback. On the contrary, we want to implement a system that is capable of detecting known objects from the training stage, and at the same time, a new object can be added to the experiment just re-training the system.

### 4.2. Experiment 2: Traffic Signals Detection by Cascade Classifier in Simulation

**Figure 10:**
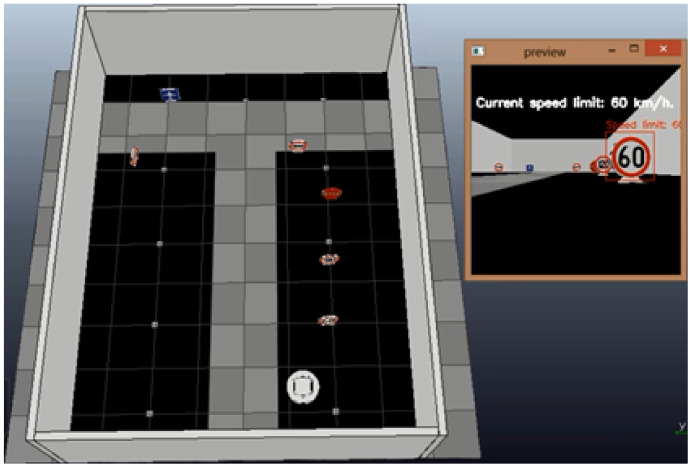
Experimentation detection of traffic signals in simulation.

After testing the system with these simple examples, the detection of more complex objects, such as traffic signals, is implemented both in simulation and with the platform. The simulation test consists of a classic example of a vehicle on a public road. The robot faces different traffic signals during its displacement. In this case, the robot must detect 6 traffic signals to reach its objective, which in this case is the airport signal. Figure 10 shows the configuration of this experiment in the V-REP simulator. On the right side of the figure, the image acquired by the robot shows this signal.

At the beginning of the experiment, the robot starts to advance linearly with both velocities at 0.0418 m/s. When it detects the first signal (60), it decreases both velocities by approximately 0.025 m/s over 4.5 s, which means that it continues advancing but with less speed. Then, it detects the 120 speed limit signal, and it increases both velocities to double the previous value to 0.05 m/s over 9.7 s and continues with a straight trajectory. After that, the next signal detected is a stop, which makes the robot decrease its speeds until reaching 0 m/s over 2.15 s.

**Figure 11:**
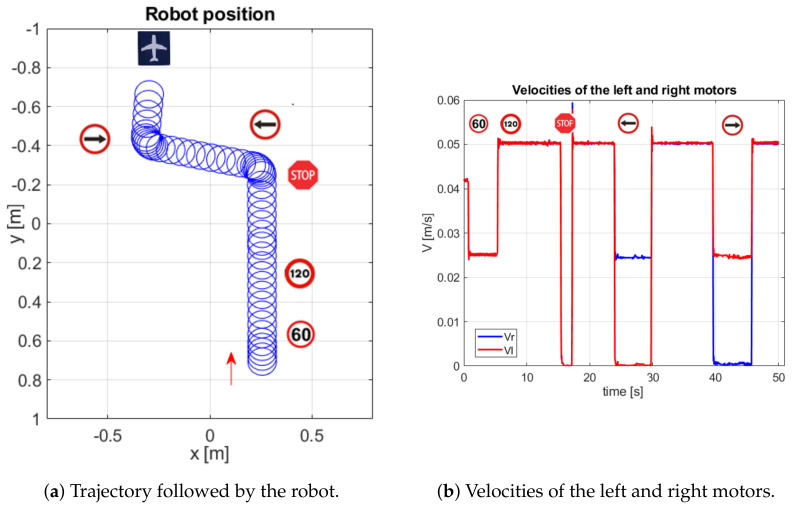
Results of the experiment in simulation environment.

The next detected signal is an arrow that indicates that the robot must turn to the left. This makes the left wheel velocity 0 m/s and the right 0.025 m/s over 5.85 s. Then, the next signal is detected by another arrow but indicates turning to the right. This makes the right velocity decrease to 0 m/s and the left velocity increase to 0.025 m/s. At this point, the last signal (airport) is detected, and the robot stops. Figure 11 shows the position of the robot during this experiment with its corresponding traffic signals in the trajectory on the left side. On the right side, the corresponding velocities of this experiment are shown.

The cascade classifier was trained by following the methodology described in Section 3.3. The training time to build the classifier for the eight traffic signals was about 5 h.

### 4.3. Experiment 3: Vision-Based Navigation in the Real Laboratory with Cascade Classifier

**Figure 12:**
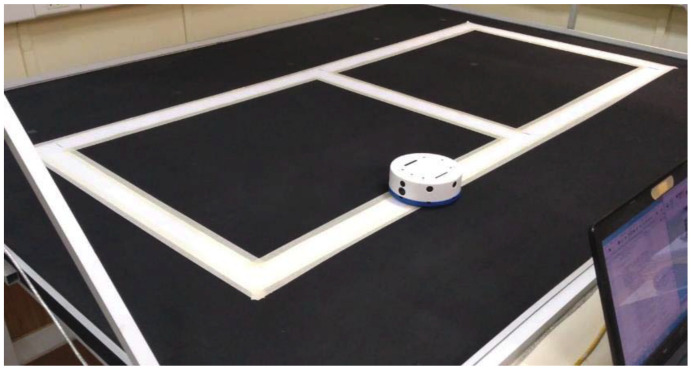
Track added to the platform to help the navigation.

In this experiment, the vision-based navigation system is implemented in the real environment to detect and classify several traffic signals. Based on the previous results, a white track was added to the real laboratory in order to aid navigation of the robot. To this end, the images of the track acquired by the on-board camera are also sent to the server and processed by the threshold technique as in [50]. Finally, the robot must move over the track by using the traffic signals. Figure 12 shows the track added to the arena.

**Figure 13:**
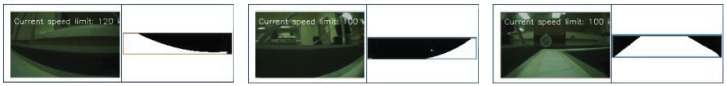
Experimental detection of tracks in a real environment.

Figure 13 shows three subfigures; in each, the left side shows the image acquired by the on-board camera of the robot, and the right side shows the result of the threshold technique applied to this image. The result of the first image is that the robot must turn to the left. The result of the second image is that the robot must turn to the right. The result of the last image is that the robot must go straight. In this way, the robot will take actions based on both the traffic signals and the track.

**Figure 14:**
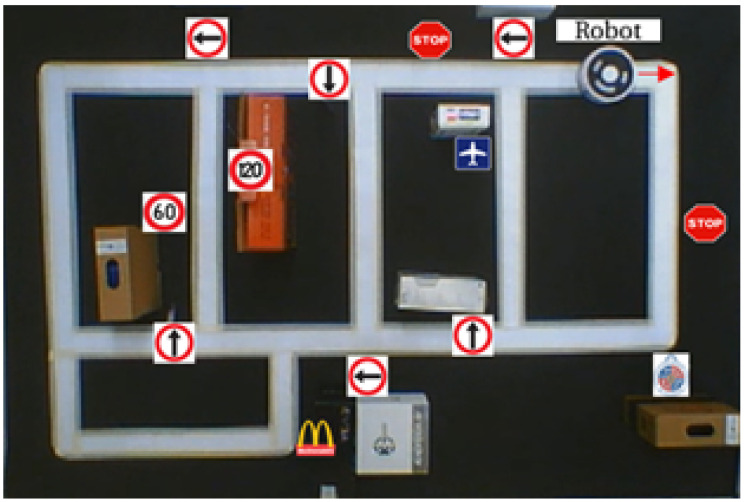
Experimental detection of traffic signals in a real environment.

After these modifications and tests, a more complex circuit was implemented with 13 traffic signals, including the logo of our university, a McDonald’s symbol and the airport symbol. Figure 14 shows the configuration of this experiment in the laboratory with the platform.

The experiment begins with the robot located above the white track to guide its path. The first manoeuvre of the robot is to turn to the right. After that, the next signal detected is a stop, which is applied to reduce the speed of the robot. The next step is a straight advance based on the track until a turn to the right is executed, also based on track information. After that, the robot advances straight along the track until a right turn arrow appears, and it continues to the target (McDonald symbol), by following the track and traffic signals.

**Figure 15:**
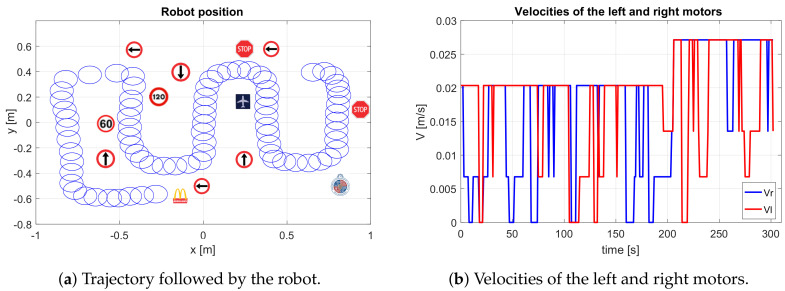
Results of the experiment developed in the real environment.

The main metric for object detection corresponds to a fraction of the frames with a successful recognition of the traffic signals. In the experiment shown in Figure 14, the detection rates for each signal are the following: 100% for right and left arrows, 72% for the stop signal and 88% for the airport signal, 62% for the 60-speed limit and 85% for the 100-speed limit signals. Figure 15 shows the results of this experiment.

On the left side, the trajectory followed by the robot in the circuit is shown; the right side shows the corresponding velocities during the experiment. In the first 195 seconds, the speeds of the motors range between 0 and 0.02 m/s since their maximum speed is equivalent to 100 km/h. Depending on the speed limit signal, the speeds of the motors increase or decrease. Thus, when the speed limit signal is 60, the maximum motor speed decreases to 0.014 m/s, and when the speed limit signal is 120, the maximum velocity increases to 0.027 m/s. The total duration of the trajectory is 300 s.

Finally, note that there are three main processing times in the real environment: (1) Acquisition and sent of the image captured by the robot’s camera to the server (approximately between 100 ms and 150 ms); (2) the classification time of the cascade algorithm (in average about 30 ms); and (3) the time required to send the command signals to the Khepera robot from the server (around 10 ms). Thus, the total processing time for the vision-based navigation system is about 200 ms, which is equivalent to 4 mm at the maximum speed of the robot (27 mm/s).

### 4.4. Experiment 4: Comparative Analysis of the Vision-Based Navigation Using Cascade Classifiers and Yolo

**Figure 16:**
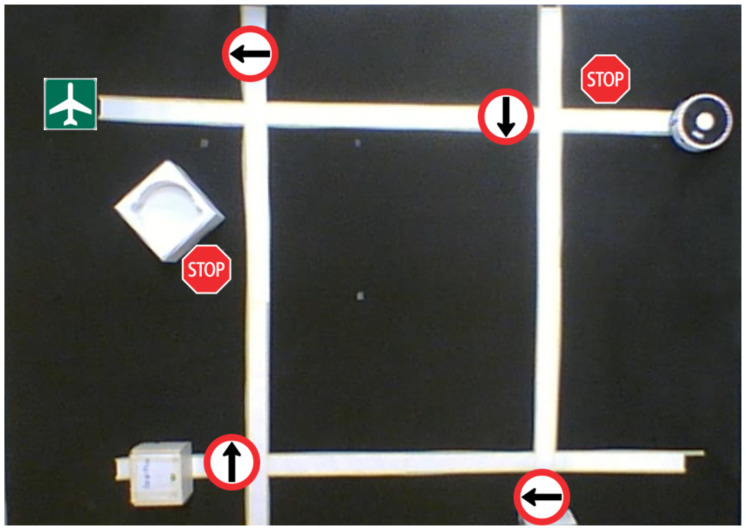
Experimental setup for the real environment.

This subsection shows the results of the comparison of the cascade classifiers and YOLO algorithm [32], which is a family of CNN (Convolutional Neural Networks) that can perform object detection in real-time. This algorithm learns a general representation of objects and outperforms several detection methods, including DPM (Deformable Parts Model) [51] and R-CNN (Region-Based Convolutional Neural Networks). In this work, the version 2 of this algorithm, also called YOLO9000 [52], has been selected. This version improves the training and performance of the original algorithm, and it can recognize up to 9000 object categories. Figure 16 shows the configuration of the implemented experiment for both algorithms (Cascade classifiers and YOLO).

**Figure 17:**
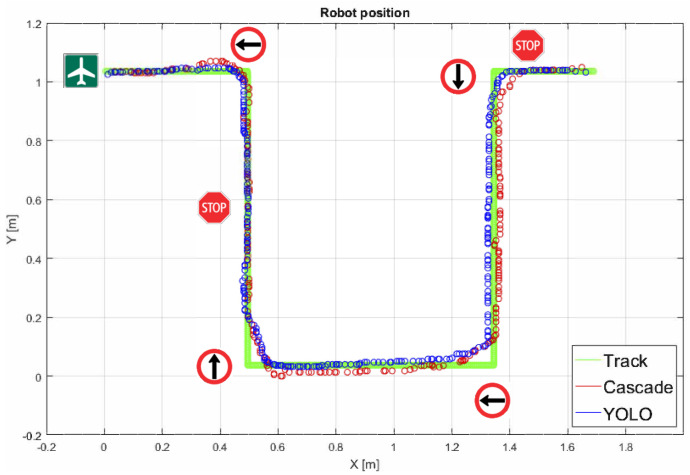
Trajectory followed by the robot for the Cascade and You Only Look Once (YOLO) algorithms.

The experiment begins with the robot on the upper right side of the figure and moving straight along the track according to the traffic signals it detects on its way. The sequence of the traffic signals detected by the robot are the following: Stop, Left arrow, Right arrow, Right arrow, Stop, Left arrow, and Airport, which is the target point. Figure 17 shows the trajectory followed by the robot for both algorithms (cascade classifiers and YOLO). The green line represents the track. The red line represents the results of the cascade algorithm and the blue line represents the results of the YOLO algorithm. As can be seen, the results are very similar.

Table 1 shows the results of the classification process for the cascade classifiers. These results show that the left and right arrows are detected and classified with a 100% success rate. The STOP signal is classified with a 72% success and the Airport signal with 88% success rate. In general, the success rate of the algorithm is 84%, which means that in 209 of 249 frames the objects are correctly recognized.

Table 2 shows the results of the same experiment, but using the YOLO algorithm. The specific success rates for each signal are the following: 99% for the left arrow, 84% for the right arrow, 97% for the STOP signal and, 52% for the Airport signal. As can be seen, the number of missed frames is 67 of 414 in total, which represents a 84% success rate. The results for both algorithms are very similar.

Finally, note that the column total frames for both algorithms represents the number of frames from the the first until the last detection of an object. This means that YOLO detects earlier the objects in the track in comparison than Cascade classifier.

## 5. Conclusions

This work presents a vision-based system in a laboratory environment. The robot used for the implementation is a Khepera IV, which presents high performance for this kind of application. However, the robot cannot carry out image processing on-board to navigate through the scenario by analysing complex information and performing object recognition, such as traffic signals on the track.

To this end, a server-side processing is added to the mobile robot platform in order to implement image processing. In this way, the system was transformed into a distributed architecture, where the image acquired by the robot is sent to the server using a WI-FI network configured for the experiments. In the server, the image is processed using different image algorithms. To perform a more complex analysis of the scenario, a cascade of classifiers has been developed to interpret several traffic signals (speed limits, yield signal, stop signal, airport, etc.). The analysis of the traffic signals allows the robot to compute the corresponding motor speeds. The calculated velocities are then sent to the robot to make the corresponding manoeuvre at each sample time. To complement the scenario, multiple tracks are added where the robot can move. The image of a track is also processed by the server.

The proposed navigation system allows to use advanced computer vision algorithms to perform sophisticated and engaging experiments with practical mobile robot laboratories for pedagogical purposes. These experiments can be tested by the students in simulation firstly, and after that they can be implemented in a real and easy-to-use environment in a relatively fast and straightforward way.

The performance of the system is highly encouraging. The communication process is fast enough to acquire the image, send it to the server and move the robot according to the velocities received. The hardest task is training the object recognition algorithms. The time can increase exponentially depending on the number of images used in the training stage. This aspect must be considered when building a similar system that includes many more objects or traffic signals. Regarding the comparison of the implemented algorithms, the results have shown that the deep learning algorithm provides an earlier detection and recognition of the object, but the success rate is very similar to the Cascade classifier.

Future work will include the implementation of different classifiers with other image processing algorithms in the server to provide the students a wide range of vision-based approaches. In addition, more robots will be included to perform exploring and collaboration navigation. A study of the relation between the speed of the robot and the image processing time will be included. Finally, as was mentioned before, the experimental platform has been developed to perform different control strategies in mobile robotics such as path following, tracking control, obstacle avoidance, and formation control. Thus, the vision-based navigation system could be combined with the others already implemented solutions to perform much more complex and realistic tasks.

## Figures and Tables

**Table 1:** Results of object detection with cascade classifier.

Signal	Total Frames	Detected Frames	Missed Frames	Success Rate
Left arrow	32	32	0	100%
Right arrow	31	31	0	100%
Stop	111	80	31	72%
Airport	75	66	9	88%
Total	249	209	40	84%

**Table 2:** Results of object detection with YOLO algorithm.

Signal	Total Frames	Detected Frames	Missed Frames	Success Rate
Left arrow	78	77	1	99%
Right arrow	129	108	21	84%
Stop	121	117	4	97%
Airport	86	45	41	52%
Total	414	347	67	84%
